# Supplementary material for: Functional interactions between neurofibromatosis tumor suppressors underlie Schwann cell tumor de-differentiation and treatment resistance
Source: Nat Commun. 2024 Jan 12;15:477. doi: 10.1038/s41467-024-44755-9 (PMC10786885; doi:10.1038/s41467-024-44755-9)
Supplement: Supplementary file 1 — Supplementary Information [file 41467_2024_44755_MOESM1_ESM.pdf]

Supplementary Figures

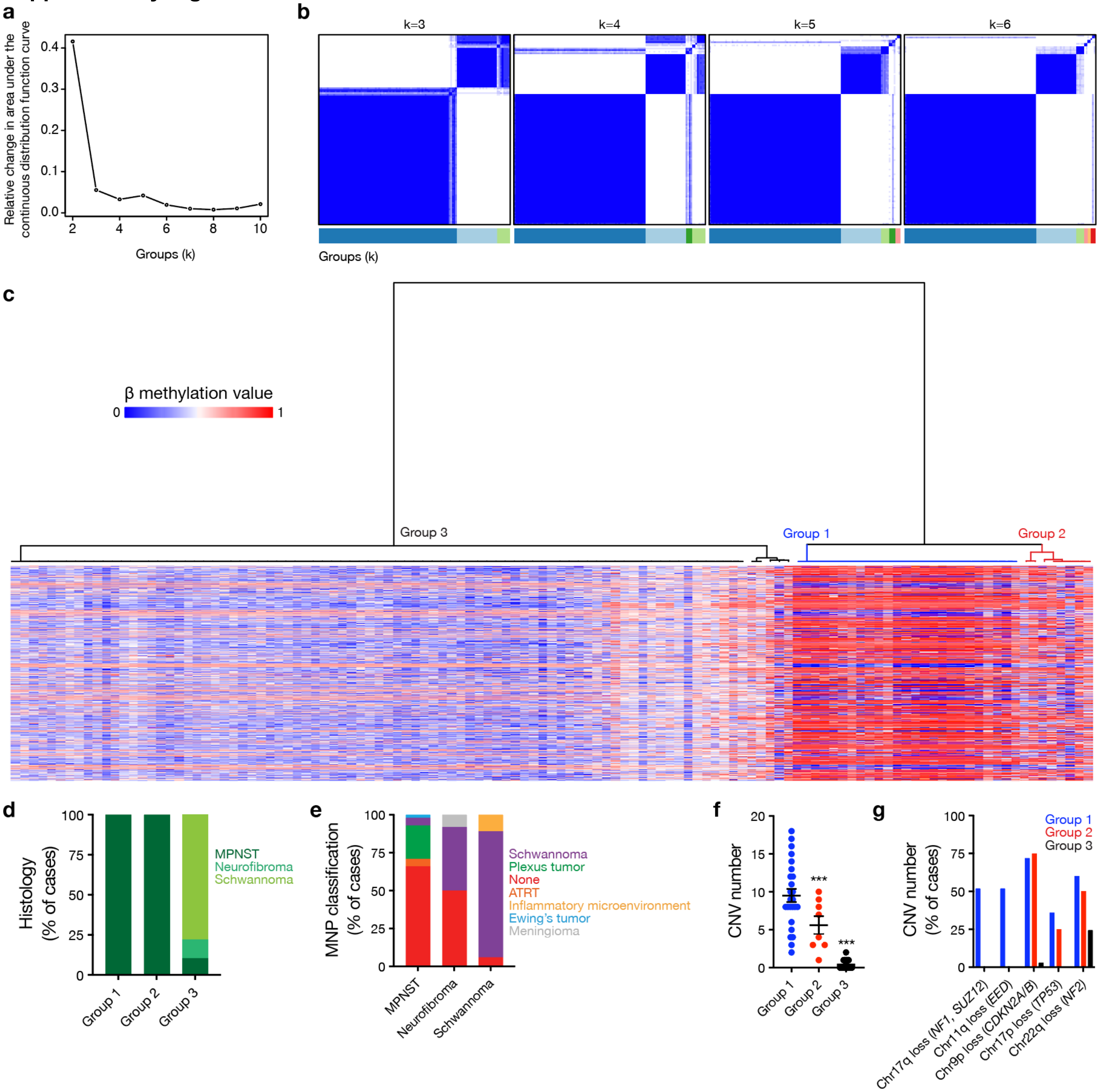

Supplementary Fig. 1. DNA methylation profiling of Schwann cell tumors. a and **b**, Consensus clustering analysis reveals stable assignment of samples to 3 DNA methylation groups across schwannomas (n=67), neurofibromas (n=10), or malignant peripheral nerve sheath tumors (MPNSTs) (n=42), with minimal relative change in CDF area under curve (AUC) with additional cluster assignments beyond 3 groups, and loss in the coherence of k means clustering beyond 3 groups. **c**, Heatmap of DNA methylation profiling k-means clustering of the 2000 most variable probes reveals 3 DNA methylation groups of Schwann cell tumors. See also Supplementary Data 1. **d**, Histological distribution across Schwann cell tumor DNA methylation groups. **e**, Molecular neuropathology classification (MNP) based on Schwann cell tumor DNA methylation profiles accurately classifies schwannomas but not neurofibromas or MPNSTs.[1] **f**, Copy number variant (CNV) number in Group 1 (n= 25) compared to Group 2 (n=8) or Group 3 (n=86) Schwann cell tumors. CNVs were derived from Schwann cell tumor DNA methylation profiles and were defined as amplifications or deletions comprising one-third or more of chromosome arms. Lines represent means. Error bars represent standard error of the means.

***p≤0.0001, Student's t tests. **g**, CNV analysis shows loss of distinct chromosome segments across Group 1, Group 2, and Group 3 tumors.

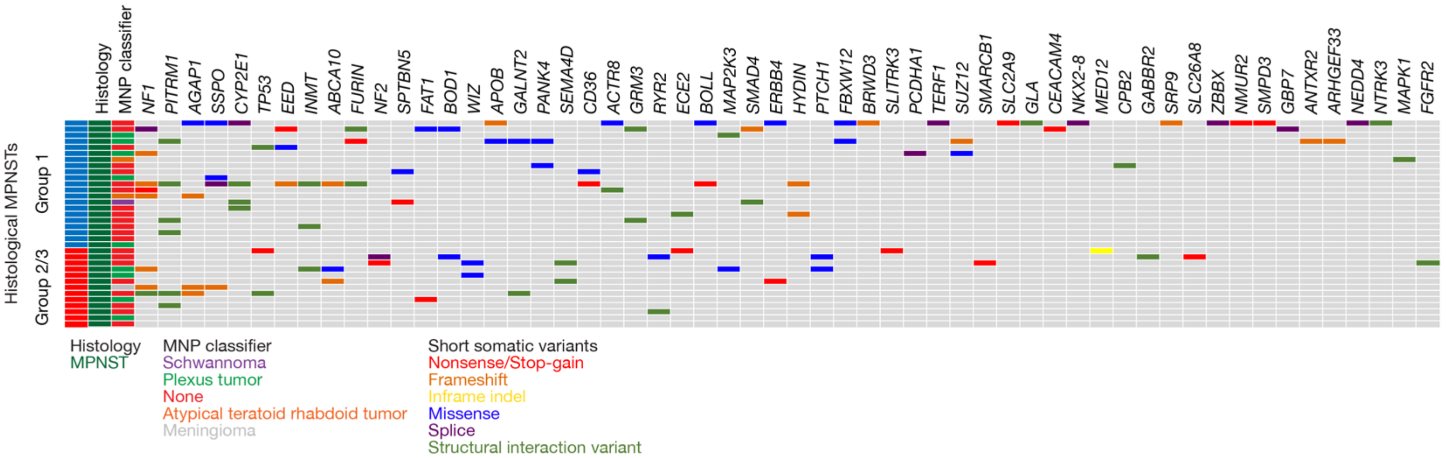

Supplementary Fig. 2. Whole exome sequencing of Group 1 or Group 2/3 histological MPNSTs. Summary matrix of short somatic variants identified in histological MPNSTs analyzed by whole exome sequencing. See also Supplementary Data 2.

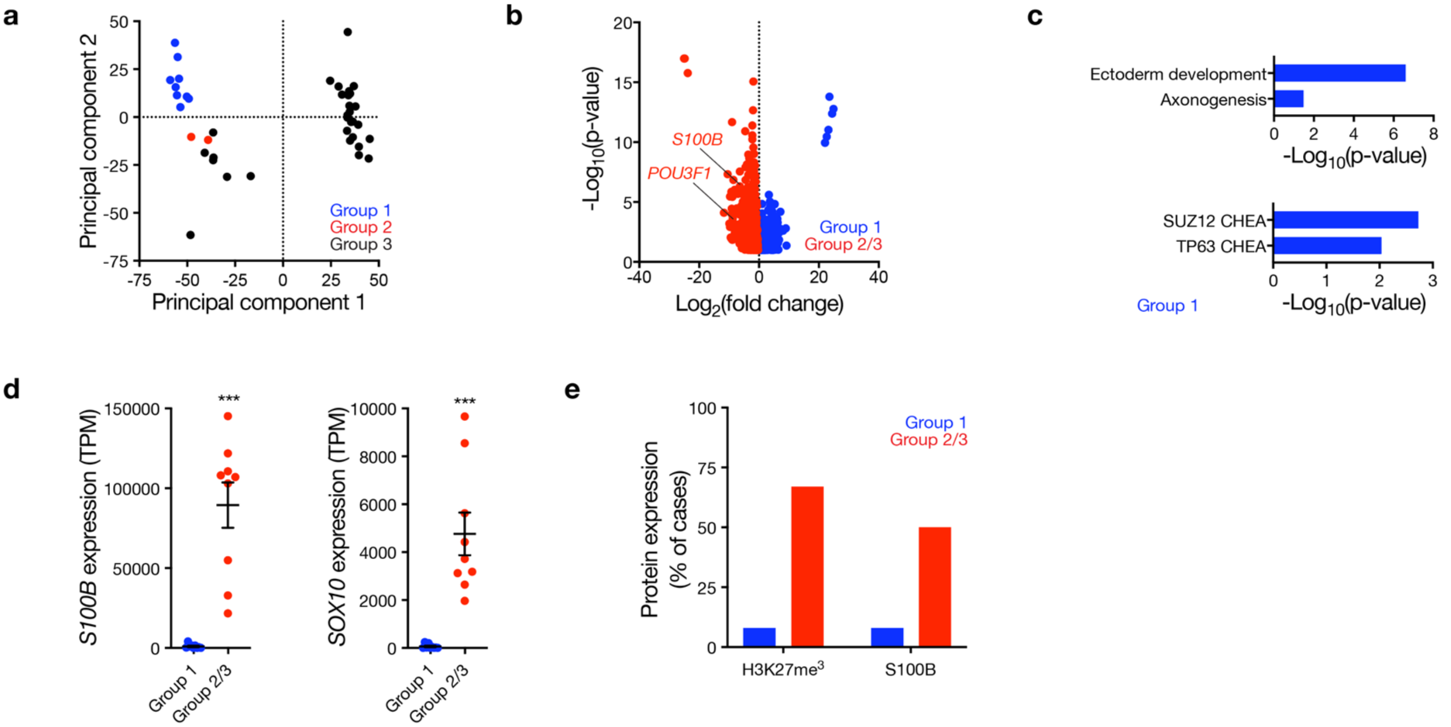

Supplementary Fig. 3. RNA sequencing and immunohistochemical analysis of Schwann cell tumors across DNA methylation groups. a, RNA sequencing and principal component analysis of Group 1 (n=9), Group 2 (n=2), or Group 3 (n=30) Schwann cell tumors separates samples. Subsequent analyses described in the Results were restricted to the 18 Group 1 or Group 2/3 histological neurofibromas and MPNSTs that underwent RNA sequencing. **b**, Volcano plot of differentially expressed genes from RNA sequencing of Group 1 (n=9) compared to Group 2/3 (n=9) histological neurofibromas and MPNSTs. The Schwann cell differentiation marker *S100B* and the positive regulator of neural differentiation *POU3F1* are annotated. **c**, Gene ontology analysis of differentially expressed genes shows enrichment of cell differentiation pathways and suppression of SUZ12 and TP63 target genes, both of which regulate cell differentiation, in Group 2/3 (n=9) compared to Group 1 (n=9) histological neurofibromas and MPNSTs. CHEA, chromatin immunoprecipitation enrichment analysis. **d**, Differential expression analysis shows suppression of Schwann cell differentiation marker genes (*S100B*, *SOX10*) in Group 1 (n=9) compared to Group 2/3 (n=9) histological neurofibromas and MPNSTs. TPM, transcripts per million. Lines represent means. Error bars represent standard error of the means. ***$p \leq 0.0001$, Student's t tests. **e**, Quantitative immunohistochemical analysis of Group 1 (n=25) or Group 2/3 (n=13) histological neurofibromas and MPNSTs shows loss of H3K27me^3 ($p<0.0001$) or S100B ($p=0.0049$), in Group 1 compared to Group 2/3 tumors. Fisher's exact tests. See also Supplementary Data 3.

a

Tumor	Histology	DNA methylation group	C0	C1	C2	C3	C4	C5	C6	C7	C8	C9	C10	C11	C12	C13	C14	C15	C16	C17	Total
3	MPNST	1	399	634	170	386	342	160	115	196	120	90	115	31	5	26	0	165	3	0	2957
43	MPNST	1	110	326	86	162	342	93	32	65	122	347	302	9	14	50	174	1	70	0	2305
47	MPNST	1	782	611	324	308	751	133	64	103	38	414	30	30	13	52	0	0	23	1	3677
12	Neurofibroma	3	511	226	768	541	230	625	287	155	250	11	170	19	102	97	0	0	4	11	4007
15	Neurofibroma	3	468	206	626	416	184	410	771	155	228	4	70	242	134	29	0	0	4	77	4024
16	Neurofibroma	3	479	194	108	263	126	376	214	263	131	8	51	35	33	23	0	1	1	0	2306
		Total	2749	2197	2082	2076	1975	1797	1483	937	889	874	738	366	301	277	174	167	105	89	19276

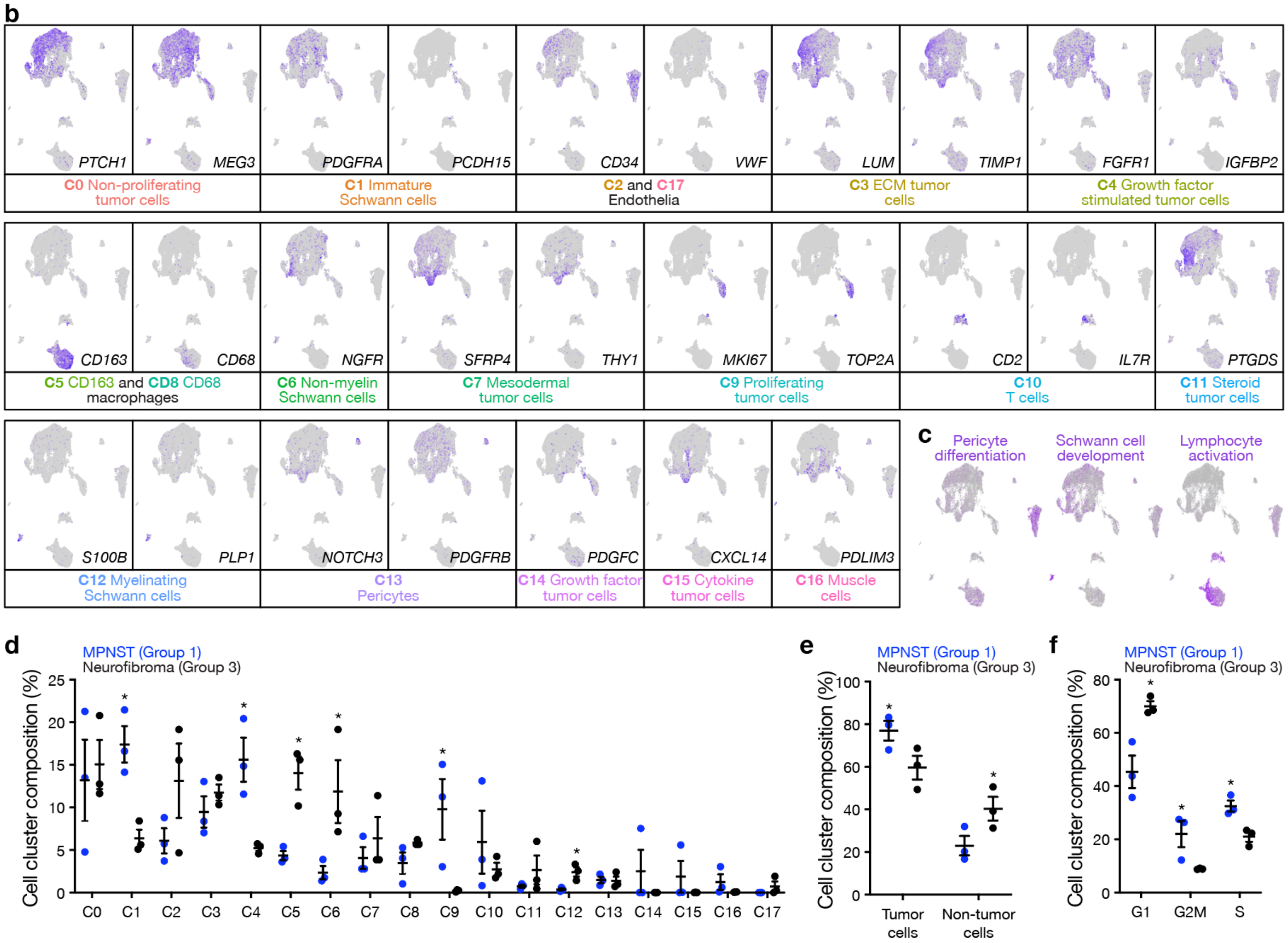

Supplementary Fig. 4. Single-nuclear RNA sequencing of Group 1 MPNSTs or Group 3 neurofibromas. **a**, Single-nuclear counts across UMAP clusters from each human Schwann cell tumor analyzed using single-nuclear RNA sequencing. Tumor histology and DNA methylation groups are annotated. A total of n=3 independent biologic samples were analyzed for each treatment group. **b**, Feature plots for cluster marker genes of harmonized Group 1 and Group 3 Schwann cell tumor single-nuclear RNA sequencing. Non-proliferating tumor cells (C0) were identified by expression of the negative regulator of Hedgehog pathway signaling *PTCH1*[2] and the p53 associated transcript *MEG3*[3,4], both of which repress proliferation in cancer. Immature Schwann cells (C1) were identified using SCtype automated classification[5], and this cluster was distinguished by expression of *PDGFRA* and *PCDH15*[20]. Endothelia (C2, C17) were identified by expression of *CD34* and *VWF*, and pericytes (C13) were identified by expression of *NOTCH3* and *PDGFRB*. Extracellular matrix (ECM) tumor cells (C3) were enriched for expression of *LUM,* an extracellular proteoglycan that binds collagens, and *TIMP1,* a metallopeptidase inhibitor that counteracts matrix metalloproteinases in the ECM. Growth factor stimulated tumor cells (C4) were identified by expression of the receptor tyrosine kinase *FGFR1* and the downstream adapter protein *IGFBP2.* Macrophages (C5, C8) were distinguished by expression of *CD163* or *CD68*. Non-myelinating Schwann cells (C6) were identified by the receptor tyrosine kinase *NGFR* expression[6], while myelinating Schwann cells (C12) were marked by expression of the differentiated Schwann cell markers *S100B* and *PLP1*. Mesodermal tumor cells (C7) were distinguished by expression of the Wnt signaling component *SFRP4* and the stem cell marker *THY1*[7], proliferating tumor cells (C9) were enriched in *TOP2A* and *MKI67* expression, and T-cells (C10) were marked by expression of *CD2* and *IL7R*. C14-17 were each limited to a single tumor sample and were defined by cluster-specific markers, as annotated. **c**, MSigDB gene set signatures for

pericyte differentiation, Schwann cell development, or lymphocyte activation validate non-tumor cell cluster definitions. **d**, Cluster composition based on DNA methylation Group 1 versus Group 3 Schwann cell tumors of origin. **e**, Group 1 Schwann cell tumors have significantly more tumor cells and significantly fewer non-tumor cells from single-nuclear RNA sequencing. **f**, Group 1 Schwann cell tumors have significantly more actively cycling cells, as shown by increased G2M and S phase cell cycle assignment from single-nuclear RNA sequencing. Lines represent means. Error bars represent standard error of the means. $^*p \leq 0.05$, Student's t tests. See also Supplementary Data 4.

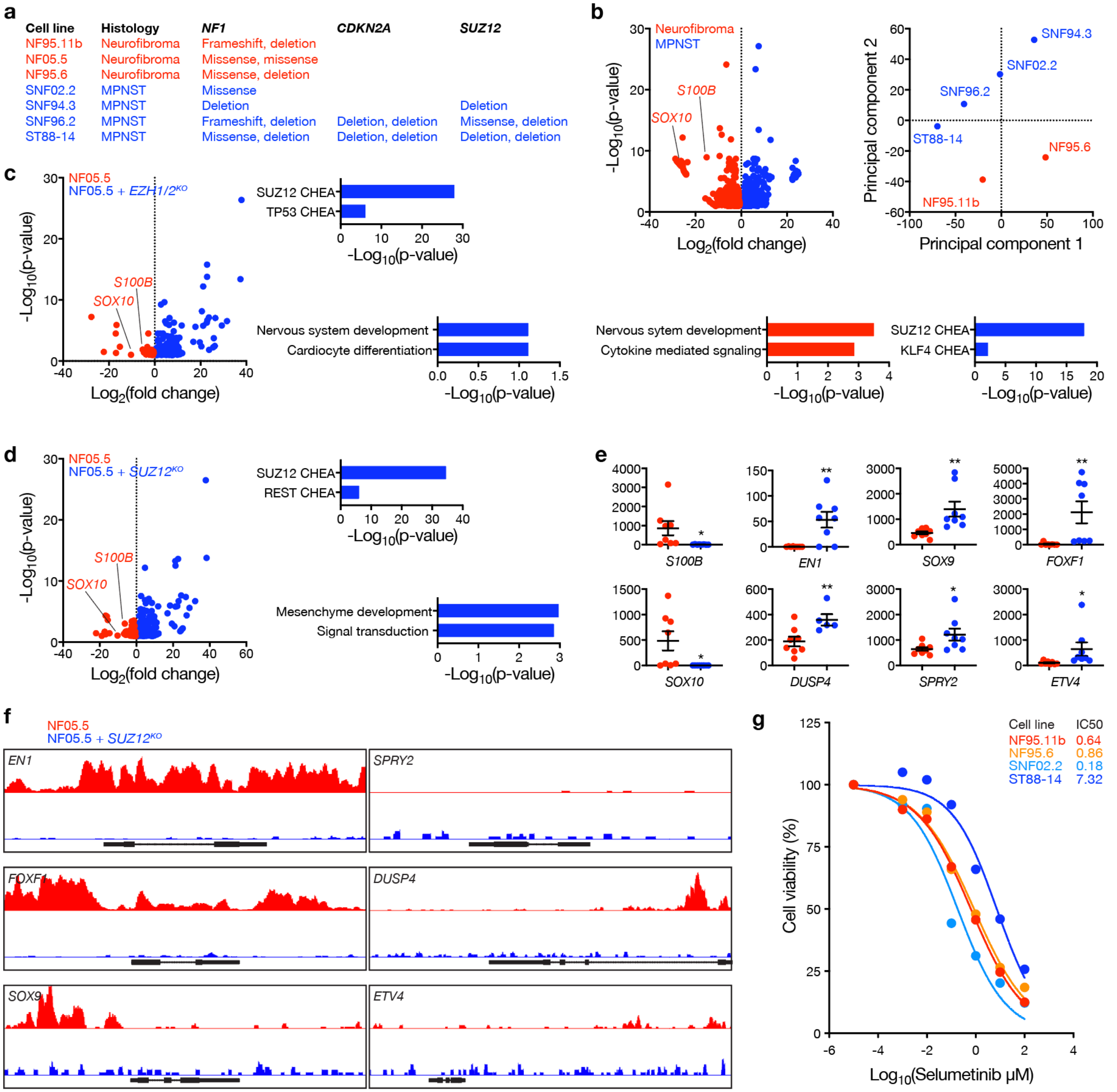

Supplementary Fig. 5. Transcriptomic, epigenomic, biochemical, and pharmacologic analysis of patient derived neurofibroma or MPNST cells. a, Patient derived neurofibroma or MPNST cells harbor *NF1, CDNK2A,* or *SUZ12* mutations affecting one or both alleles[8]*.* All three neurofibroma cell lines harbored biallelic *NF1* loss. For the MPNST cell lines, SNF02.2 harbored an *NF1* missense mutation, SNF94.3 harbored *NF1* and *SUZ12* mutations, and both SNF96.2 and ST88-14 cells harbored loss of *NF1, CDKN2A,* and *SUZ12*. **b**, RNA sequencing volcano plot from differential expression analysis, principal component analysis, and gene ontology analyses reveal neurofibroma and MPNST cells are distinguished by enrichment of cell differentiation pathways or de-repression of SUZ12 or the master cell fate regulator KLF4 target genes, respectively. CHEA, chromatin immunoprecipitation enrichment analysis. **c**, RNA sequencing differential expression and gene ontology analyses show simultaneous CRISPR deletion of both PRC2 epigenetic complex members *EZH1* and *EZH2*[9], which can functionally compensate for one another, induces PRC2 target genes and regulators of cell differentiation in neurofibroma cells. **d**, RNA sequencing differential expression and gene ontology analyses show deletion of the obligate PRC2 epigenetic complex member *SUZ12*[9] induces PRC2 target genes and regulators of cell differentiation in neurofibroma cells enriched for modulating expression of SUZ12 and the

epigenetic regulator REST target genes. **e**, RNA sequencing transcripts per million (TPM) expression shows suppression of Schwann cell differentiation markers (*S100B, SOX10*), enrichment of canonical Ras target genes (*DUSP4, SPRY2, ETV4*), or enrichment of neural crest development genes (*EN1, SOX9, FOXF1)* in PRC2-mutant compared to PRC2-intact Schwann tumor cells. PRC2-mutant (blue): ST88-14, SNF02.2, SNF94.3, SNF96.2, or NF05.5 cells with *SUZ12* or simultaneous *EZH1* and *EZH2* knockout (n=8). PRC2-intact (red): NF9511b, NF95.6, and NF05.5 cells ± *EZH1* or *EZH2* knockout (n=8). Lines represent means. Error bars represent standard error of the means. *p<0.05, ***p≤0.0001, Student's t tests. **f**, Integration of H3K27me[3] ChIP sequencing data from PRC2-intact versus PRC2-mutant neurofibroma cells[9] with RNA sequencing data across neurofibroma and MPNST cells suggests direct epigenetic de-repression of development genes (*EN1, SOX9, FOXF1)*, and indirect induction of Ras target genes (*DUSP6, SPRY2, ETV4*) in PRC2-mutant compared to PRC2-intact cells. **g**, Selumetinib dose-response viability curves for neurofibroma or MPNST cells following 48 hours of treatment reveals ST88-14 MPNST cells with bi-allelic *SUZ12* mutations are resistant to MEK inhibition while PRC2-intact SNF0.2 MPNST cells and both neurofibroma cell lines remain sensitive to selumetinib. See also Supplementary Data 5.

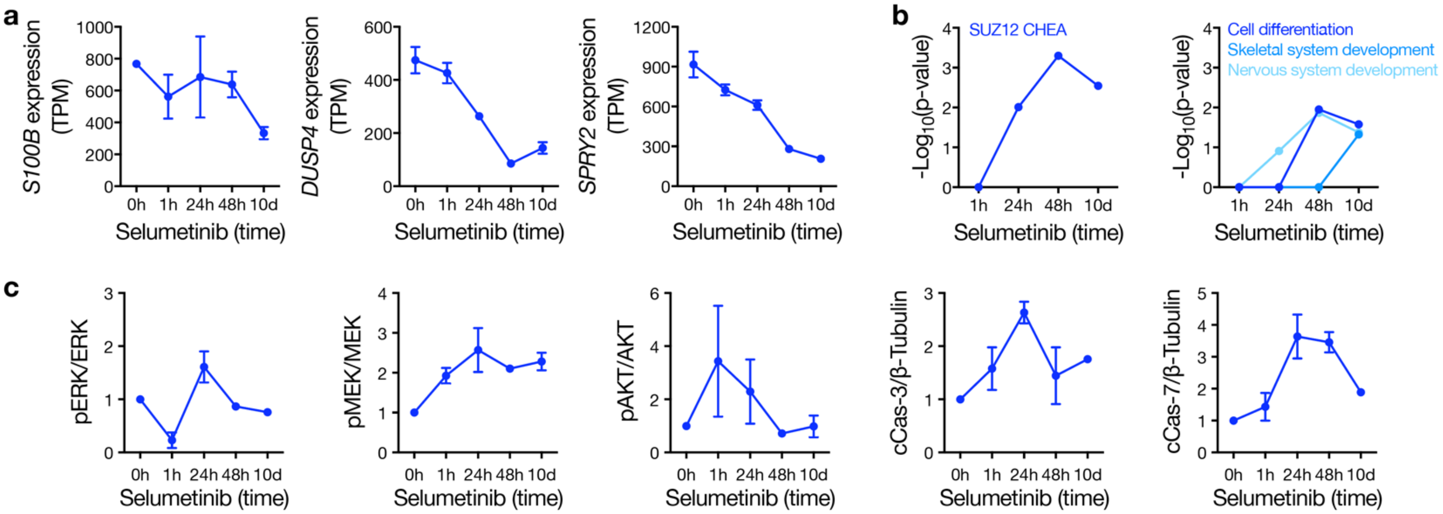

Supplementary Fig. 6. **Transcriptomic and biochemical analyses of selumetinib responses over time in neurofibroma cells**. **a**, RNA sequencing of NF95.11b neurofibroma cells after treatment with 1 μM selumetinib or vehicle (n=2 independent biologic experiments per condition or timepoint) control demonstrates selumetinib inhibits expression of the Schwann cell differentiation marker *S100B* and Ras target genes *DUSP4* and *SPRY2*. TPM, transcripts per million. **b**, RNA sequencing and gene ontology analyses of NF95.11b neurofibroma cells after treatment with 1 μM selumetinib shows selumetinib treatment inhibits cell differentiation programs of neural crest derived lineages such as the nervous or skeletal systems. CHEA, chromatin immunoprecipitation enrichment analysis. See also Supplementary Data 6. **c**, Quantification of immunoblots measuring pERK, pMEK, pAkt, cleaved caspase 3 (cCas-3), or cleaved caspase 7 (cCas-7) after treatment with 1 μM selumetinib. Phospho-proteins were normalized to total protein levels. CC7 and CC3 were normalized to β-Tubulin (n=2 independent biologic experiments per condition or timepoint). See also Fig. 2c.

a

JW23.2 tumor	Treatment	C0	C1	C2	Total
1	Vehicle	5093	3640	2637	11370
2	Vehicle	2589	1867	1048	5504
3	Selumetinib	1850	740	570	3160
4	Selumetinib	1594	621	791	3006
5	Selumetinib	1920	901	747	3568
	Total	13046	7769	5793	26608

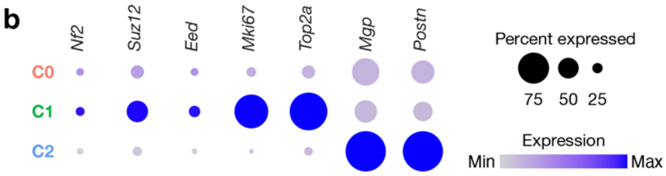

Supplementary Fig. 7. Single-cell RNA sequencing of selumetinib responses in MPNST allografts. a, Single-cell counts across UMAP clusters from each JW23.3 MPNST allograph analyzed using single-cell RNA sequencing. C0, selumetinib resistant cells. C1, proliferating tumor cells. C2, differentiated tumor cells. **b**, Expression of the cell proliferation markers *Mki67* and *Top2a* were enriched in proliferating C1 cells, while expression of the differentiation markers *Mgp* and *Postn* were enriched in C2 differentiated cells. See also Supplementary Data 7.

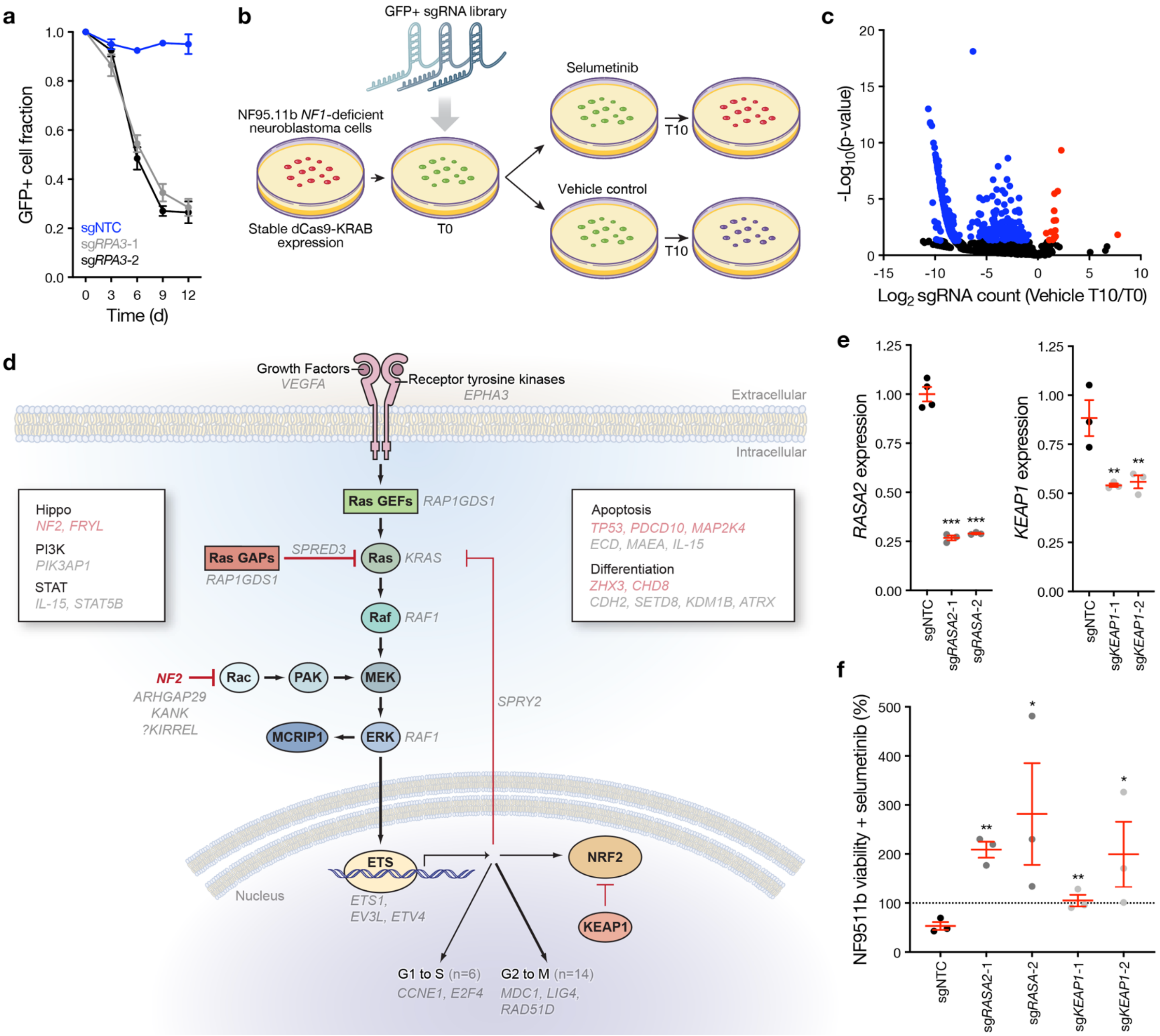

Supplementary Fig. 8. A genome wide CRISPRi screen identifies mechanisms driving selumetinib responses in neurofibroma cells. **a**, CRISPRi suppression of the common essential gene *RPA3* leads to decreased *NF1*-mutant NF95.11b neurofibroma cell fitness as measured by GFP+ cell fraction compared to non-targeted control sgRNA (sgNTC) over time, validating the functionality of CRISPRi machinery in NF95.11b cells (n=2 independent biologic replicates per sgRNA). **b**, Schematic of genome-wide CRISPRi screen design. **c**, Distribution of core essential genes (sgRNA abundance in vehicle conditions at day 10 compared to T0) shown as a volcano plot from triplicate genome-wide CRISPRi screens in NF95.11b neurofibroma cells stably expressing dCas9-KRAB and treated with DMSO vehicle control for 10 days. 493 Cancer DepMap core essential genes were significantly depleted (blue) and 19 were significantly enriched (red), supporting the specificity of CRISPRi screen results. X-axis is normalized log2 sgRNA abundance count. Black shows core essential genes not meeting filtering criteria for significantly depleted or enriched hits. **d**, Schematic of CRISPRi screen hits mediating selumetinib resistance (blue hues, loss leads to selumetinib sensitivity; red hues, loss leads to selumetinib resistance). **e**, QPCR validation of CRISPRi suppression of the screen hits *RASA2* and *KEAP1* in NF95.11b cells (n=3 biologic replicates per sgRNA). **f**, Validation that suppression of the selumetinib resistance hits *RASA2* and *KEAP1* sensitizes NF95.11b cells to selumetinib (n=3 biologic replicates per sgRNA). Lines represent means. Error bars represent standard error of the means. *p<0.05, **p≤0.01, ***p≤0.0001, Student's t tests. See also Supplementary Data 8.

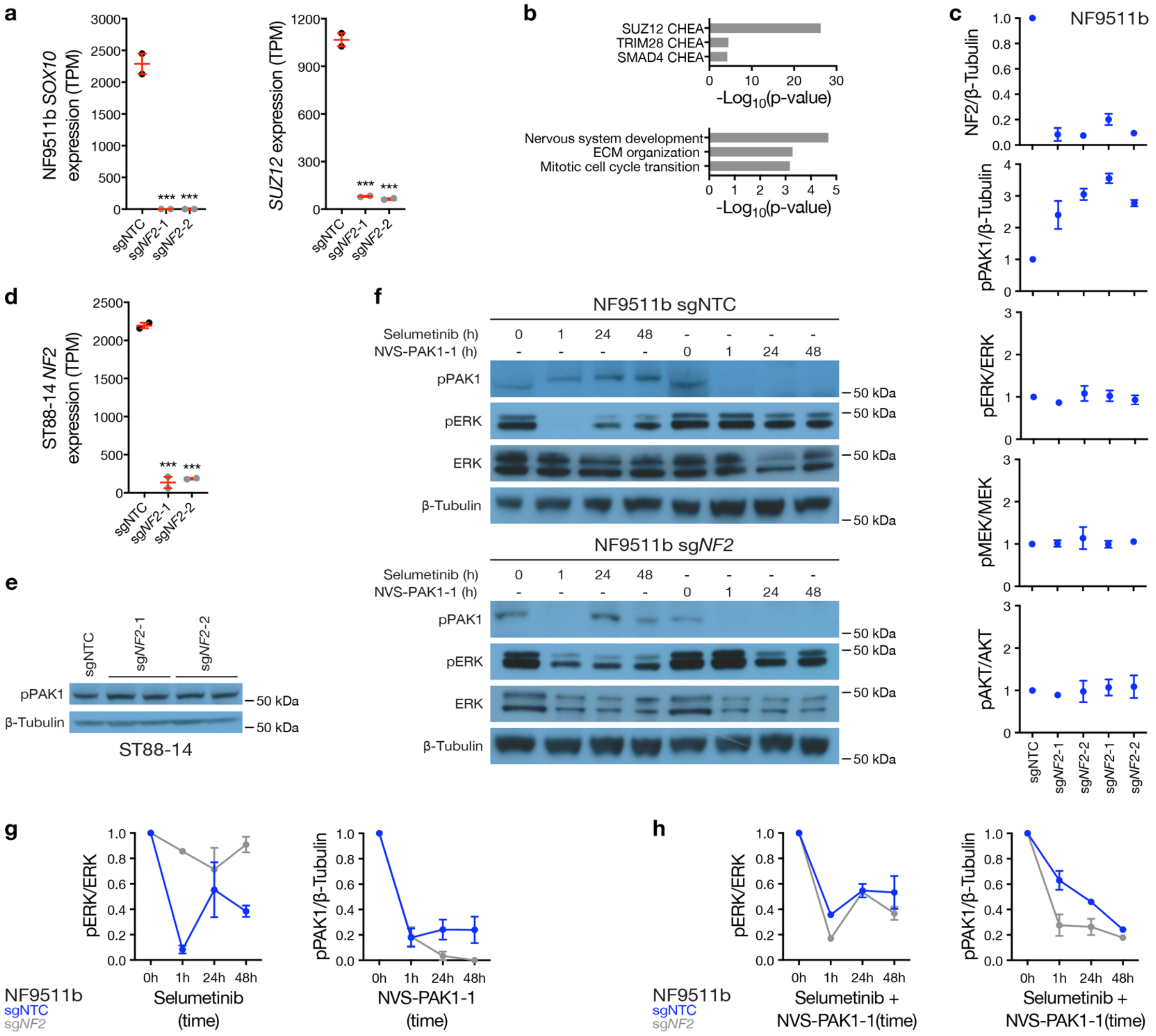

Supplementary Fig. 9. *NF2* CRISPRi suppression and pharmacologic responses in *NF1*-mutant NF95.11b neurofibroma or ST88-14 MPNST cells. a, RNA sequencing of *NF1*-mutant NF95.11b neurofibroma cells stably expressing dCas9-KRAB and transduced with non-targeting control sgRNAs (sgNTC) or 2 separate sgRNAs suppressing *NF2* (n=2 independent biologic replicates per sgRNA) reveals *NF2* suppression inhibits expression of Schwann cell differentiation marker genes (*SOX10, SUZ12*). TPM, transcripts per million. See also Supplementary Data 9. **b**, Gene ontology analysis of differentially expressed genes shows enrichment of SUZ12 targets, targets of the differentiation transcription factor *TRIM28* (encoding the protein KAP1), and targets of the tumor suppressor transcription factor *SMAD4* after *NF2* CRISPRi suppression of *NF2* in NF95.11b neurofibroma cells. **c**, Quantification of immunoblots evaluating NF2, pPAK1, pERK, pMEK, or pAKT following CRISPRi suppression of *NF2* in *NF1*-mutant NF.9511b cells (n=2 independent biologic replicates per timepoint). See also Fig. 3c. **d**, CRISPRi suppression of *NF2* in *NF1*-mutant, *SUZ12*-mutant ST88-14 MPNST cells. See also Supplementary Data 10. **e**, *NF2*-deficient ST88-14 MPNST cells do not demonstrate further induction of pPAK1 compared to cells expressing sgNTC (n=2 biologic replicates per condition). **f,** Treatment with selumetinib or the PAK1 inhibitor NVS-PAK1-1 in *NF2*-intact versus *NF2*-deficient neurofibroma NF.9511b cells (n=3 biologic replicates per condition). **g**, Quantification of immunoblots evaluating pERK or pPAK1 following selumetinib of NVS-PAK1-1 monotherapy (n=2 independent biologic replicates per timepoint). **h**, Quantification of immunoblots evaluating pERK or pPAK1 following combined selumetinib and NVS-PAK1-1 dual inhibitor therapy (n=2

independent biologic replicates per timepoint). See also Fig. 3d. Lines represent means. Error bars represent standard error of the means. *$p \leq 0.05$, **$p \leq 0.01$, ***$p \leq 0.0001$, Student's t tests.

Uncropped immunoblots for Supplementary Fig. 9.

9e
pPAK1 (middle band is PAK1, other bands are alternate PAK proteins expressed in ST88-14 cells)

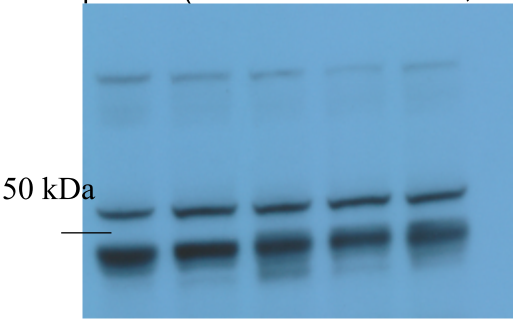

bTub

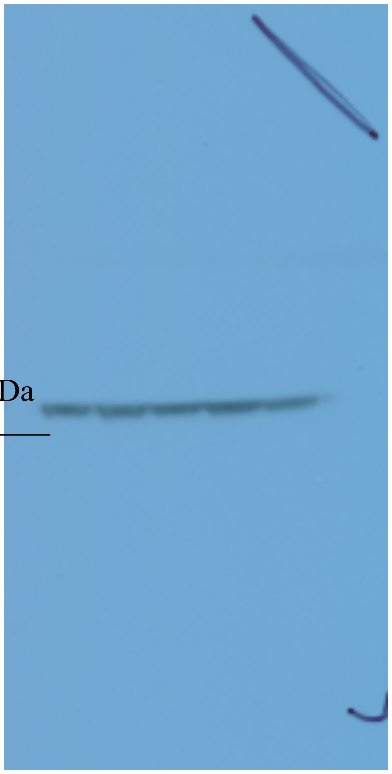

9f
sgNTC treated with selumetinib or NVS PAK1-1 monotherapy

pPAK1 (lower band is pPAK1, upper is PAK2)

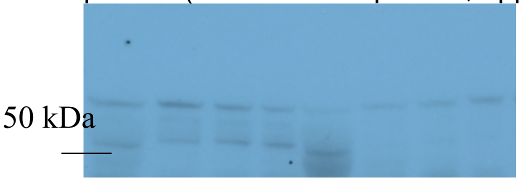

pERK

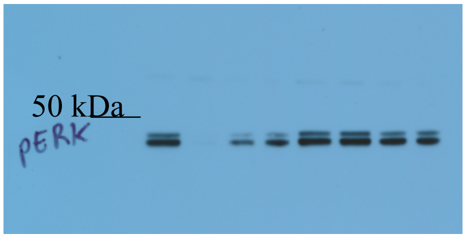

tERK

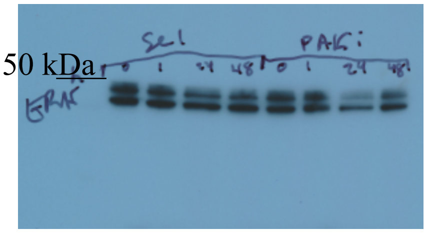

bTub

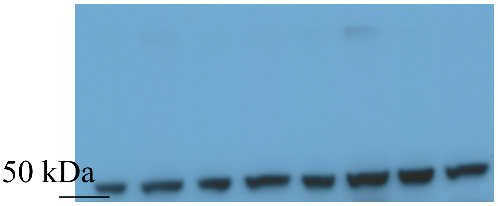

sgNF2 treated with selumetinib or NVS PAK1-1 monotherapy

pPAK1 (middle band is pPAK1)

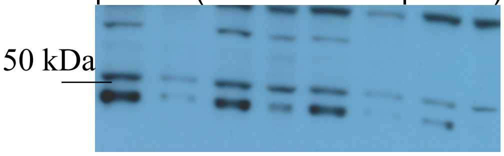

pERK

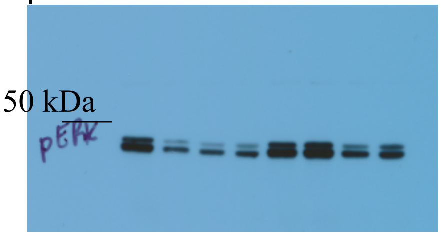

tERK

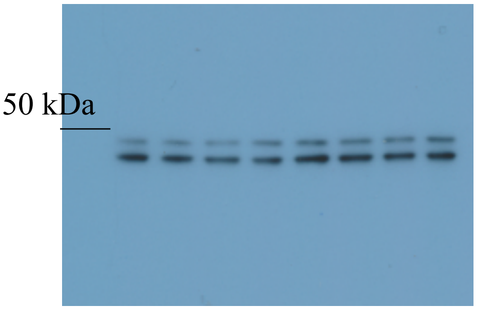

bTub (upper band; lower bands are pERK probed on same film to ensure accurate loading)

50 kDa

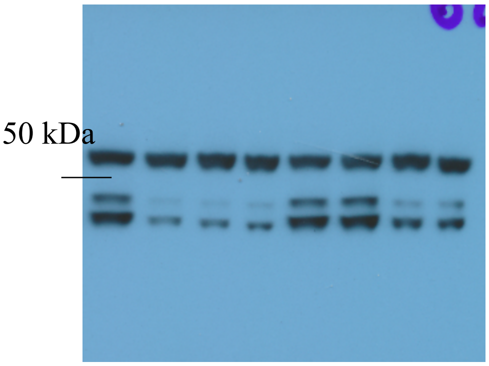

Supplementary Reference List

1. Capper, Jones, S. DNA methylation-based classification of central nervous system tumours. *Nature* **555**, 469–474 (2018).
2. Pak, E. & Segal, R. A. Hedgehog Signal Transduction: Key Players, Oncogenic Drivers, and Cancer Therapy. *Dev Cell* **38**, 333–344 (2016).
3. Zhou, Y. *et al.* Activation of p53 by MEG3 non-coding RNA. *Journal of Biological Chemistry* **282**, 24731–24742 (2007).
4. Zhu, J. *et al.* Long noncoding RNA MEG3 interacts with p53 protein and regulates partial p53 target genes in hepatoma cells. *PLoS One* **10**, 1–15 (2015).
5. Ianevski, A., Giri, A. K. & Aittokallio, T. Fully-automated and ultra-fast cell-type identification using specific marker combinations from single-cell transcriptomic data. *Nat Commun* **13**, 1–10 (2022).
6. Vasudevan, H. N., Lucas, C. H. G., Villanueva-Meyer, J. E., Theodosopoulos, P. V. & Raleigh, D. R. Genetic Events and Signaling Mechanisms Underlying Schwann Cell Fate in Development and Cancer. *Neurosurgery* **88**, 234–245 (2021).
7. Koren, E. *et al.* Thy1 marks a distinct population of slow-cycling stem cells in the mouse epidermis. *Nat Commun* **13**, 1–16 (2022).
8. Li, H., Chang, L. J., Neubauer, D. R., Muir, D. F. & Wallace, M. R. Immortalization of human normal and NF1 neurofibroma Schwann cells. *Laboratory Investigation* **96**, 1105–1115 (2016).
9. Wassef, M. *et al.* EZH1/2 function mostly within canonical PRC2 and exhibit proliferation-dependent redundancy that shapes mutational signatures in cancer. (2019) doi:10.1073/pnas.1814634116.
