# Supplementary material for: Functional interactions between neurofibromatosis tumor suppressors underlie Schwann cell tumor de-differentiation and treatment resistance
Source: Nat Commun. 2024 Jan 12;15:477. doi: 10.1038/s41467-024-44755-9 (PMC10786885; doi:10.1038/s41467-024-44755-9)
Supplement: Supplementary file 3 — Description of Additional Supplementary Files [file 41467_2024_44755_MOESM3_ESM.pdf]

Description of Additional Supplementary Files

File Name: Supplementary Data 1
Description: Top 2000 most variable DNA methylation probe beta values across 119 Schwann cell tumors.

File Name: Supplementary Data 2
Description: Whole exome sequencing of short somatic variants from Group 1 or Group 2 Schwann cell tumors.

File Name: Supplementary Data 3
Description: RNA sequencing transcripts per million expression matrix from Group 1, 2, or 3 Schwann cell tumors.

File Name: Supplementary Data 4
Description: Cell cluster marker genes from single-nuclear RNA sequencing of MPNST (Group 1) or benign neurofibroma (Group 3) NF1-mutant Schwann cell tumors. Statistical analysis was performed in Seurat using a Bonferroni corrected two sided Wilcoxon rank sum test

File Name: Supplementary Data 5
Description: RNA sequencing transcripts per million expression matrix from patient-derived neurofibroma or MPNST cells.

File Name: Supplementary Data 6
Description: RNA sequencing transcripts per million expression matrix from neurofibroma cells treated with selumetinib versus vehicle control.

File Name: Supplementary Data 7
Description: Cell cluster marker genes from single-cell RNA sequencing of JW23.3 MPNST allografts treated with selumetinib versus vehicle control. Statistical analysis was performed in Seurat using a Bonferroni corrected two sided Wilcoxon rank sum test

File Name: Supplementary Data 8
Description: Raw CRISPRi screen sequencing read counts per sgRNA vector per replicate. Statistical analysis was performed using a Bonferroni corrected two sided Wald test.

File Name: Supplementary Data 9
Description: RNA sequencing transcripts per million expression matrix from neurofibroma cells after CRISPRi suppression of NF2 versus non-targeting control sgRNAs.

File Name: Supplementary Data 10
Description: RNA sequencing transcripts per million expression matrix from MPNST cells after CRISPRi suppression of NF2 versus non-targeting control sgRNAs.

File Name: Supplementary Data 11
Description: RNA sequencing analysis of core Hippo components or transcriptional target gene signature following NF2 loss in neurofibroma cells
